# Challenges of Health Data Use in Multidisciplinary Chronic Disease Care: Perspective from Heart Failure Care

**DOI:** 10.3390/jcdd10120486

**Published:** 2023-12-05

**Authors:** Pupalan Iyngkaran, Wania Usmani, Fahad Hanna, Maximilian de Courten

**Affiliations:** 1 Department of Health Sciences, Torrens University Australia, Melbourne 3000, Australia; wania.usmani@health.torrens.edu.au; 2 Public Health Program, Department of Health and Education, Torrens University Australia, Melbourne 3000, Australia; fahad.hanna@torrens.edu.au; 3 Mitchell Institute for Health and Education Policy, Victoria University, Melbourne 3000, Australia; maximilian.decourten@vu.edu.au

**Keywords:** chronic disease management, health data, congestive heart failure, guidelines, processes of care

## Abstract

The healthcare sector generates approximately 30% of all the world’s data volume, mostly for record keeping, compliance and regulatory requirements, and patient care. Healthcare data often exist in silos or on different systems and platforms due to decentralised storage and data protection laws, limiting accessibility for health service research. Thus, both the lack of access to data and more importantly the inability to control data quality and explore post-trial (phase IV) data or data with translational relevance have an impact on optimising care and research of congestive heart failure (CHF). We highlight that for some diseases, such as CHF, generating non-traditional data has significant importance, but is hindered by the logistics of accessing chronic disease data from separate health silos and by various levels of data quality. Modern multidisciplinary healthcare management of cardiovascular diseases—especially when spanning across community hubs to tertiary healthcare centres—increases the complexities involved between data privacy and access to data for healthcare and health service research. We call for an increased ability to leverage health data across systems, devices, and countries.

## 1. Introduction

The healthcare sector generates a large amount of data. According to RBC Capital, approximately 30% of all the world’s data volume is now being generated by the healthcare industry and by 2025, the annual growth rate for health data will be 36%, mostly for record keeping, compliance and regulatory requirements, and patient care [1]. Nonetheless, healthcare data can exist in silos or on different systems and platforms due to decentralised storage and data protection laws, limiting accessibility for health service research [2].

Thus, this perspective focuses on the lack of access to data and more importantly the inability to control data quality and explore post-trial (phase IV) data or data with translational relevance with reference to congestive heart failure (CHF) research. We also highlight that for some diseases, such as CHF, generating non-traditional data has great importance, but is hindered by the logistics of accessing chronic disease data from separate health silos and by various levels of data quality.

We argue in this paper that modern multidisciplinary healthcare management of cardiovascular diseases—especially when spanning across community hubs to tertiary healthcare centres—increases the complexities of data privacy and access to data for healthcare and health service research. We call for an increased ability to leverage health data across systems, devices, and countries.

## 2. Traditional and Modern Data Sources, Processing, and Implications

### 2.1. Traditional Heart Failure Data

Prior to health system data, population studies focusing on heart health (e.g., the Framingham Heart Study, the Olmstead County study), clinical trials, performance improvements (OPTIMIZE-HF, GWTG-HF), and registries (e.g., ADHERE) provided quantitative data that informed clinical care and publications [3]. More recently, the importance of adding more subjective aspects of patients’ journeys was added to produce a more comprehensive picture of heart failure data.

Clinical trials make up the smallest percentage of the health industry’s activities; however, they are the basis for confirming hypotheses and translating preliminary evidence into definitive gold standard evidence. Phase III randomised trials are invariably multicentred, with hospital patients subjected to the treatment or placebo arm of a study [3]. To achieve this level of data, a high level of organisation and funding is required. In comparison, phase IV or post-translational data are generated by the broader health service industry. Importantly, by broadening the data sources, the data quality lacks rigorous control against verifiable research standards. It is also in this phase, after treatments are approved, that actual cost-effectiveness is assessed. However, in real-life or community settings where patients will undertake several measures to maintain their health in addition to the prescribed treatment, cost-effectiveness can be difficult to measure accurately, including finding appropriate control comparison groups.

### 2.2. Data Journey during the Research Phase and Post-Trial Phase

Health data are generated through research studies or clinical work. Data generation starts at leading discoveries through laboratory research to clinical trials. The largest volume of health research data is generated in phase IV. This post-trial phase occurs when proven treatments are administered at a population level. During this period, observations can also be made about how effective treatments are outside controlled trials. Reconciling the accessibility of data across these phases will be beneficial, along with an established data standard.

### 2.3. The Wide Array of Data in Clinical Heart Failure Management

Modern clinical heart failure management has evolved to include a wide array of data types. These include clinical data which can be gathered via wearables, imaging techniques, echocardiography, and electronic health records [4]. Clinical data are one of the two main data types most commonly used to study HF. A useful way to structure the different types of health data related to disease management is by using the universal standardised disease management taxonomy developed by Krumholtz, which describes eight service domains, which include the patient, recipient, intervention content, delivery personnel, method of communication, environment, and outcome measure as categories. There are also over 35 subdomains [5]. In terms of knowledge translation, individual health jurisdictions will answer questions relevant to their service, and the fragmentation of health services and policy can become more complex with fragmentation between health jurisdictions within the same system.

There are numerous silos of healthcare datasets, and patients with CHF will experience more data gaps as modern CHF care requires chronic and multidisciplinary care across the spectrum of health services from primary care to tertiary hospital admissions. The very process of compartmentalising health service provision often creates data silos. Thus, it must be reinforced that while organisations create individual administrations, this does not remove the need to retain patient data as the patient visits various parts of the health service spectrum. This requirement for data travel should also include clinical trials, which generate more data on participants with heart failure, often with little connection to health services.

To balance data privacy and access, an understanding of care delivery in complex chronic diseases is useful. With a vast network of services involved in comprehensive CHF care, quantitative CHF data alone may not be adequate for finding treatment solutions. 

### 2.4. Dimensions of Chronic Disease Care: Implications for Heart Failure Health Data

Both the complexity of HF care and its increasing prevalence provide an impetus for broadening access to health data beyond the traditional sources of data. 

Applying chronic disease care dimensions to heart failure, we note [3]:

- Chronicity: Chronic cardiovascular diseases, including CHF, make up 85% of presentations to primary care. The strength of guidelines and gaps in community outcomes confirms translational issues between evidence and community uptake;
- Complexity: CHF has a trajectory of decompensation, stability, and eventual palliation, and requires multidisciplinary care throughout the course of the illness. CHF programs are required to navigate the complexity of the disease, high resource use, and additional burdens caused by comorbid conditions. All these influence CHF outcomes. Therefore, screening, prevention, monitoring, and treatment are essential;
- Divergence: Although there have been significant advancements in therapy, chronic heart failure (CHF) remains widespread, particularly with the rise in heart failure with preserved ejection fraction (HFpEF) syndrome in the aging population. HFpEF currently constitutes over 50% of all CHF cases, especially among elderly individuals;
- Burden: It is increasingly clear that this syndrome places a significant strain on both patients and healthcare systems, even before considering the underlying causes or additional coexisting conditions. These factors are present in at least 50% of cases and include conditions such as ischemic heart disease, as well as comorbidities like diabetes, renal failure, sleep apnoea, and hypertension. This burden translates into the complexity of the disease, increased utilisation of healthcare resources, a high rate of readmissions, the need for long-term and often lifelong treatments, and regular support from multidisciplinary healthcare services;
- Heterogeneity: Risk factors including race, smoking, drug and alcohol use, and socioeconomic and other demographic factors contribute to healthcare gaps or differential distribution of health resources, e.g., geography or social isolation add sociodemographic variables to disease phenotype;
- Unmet needs: Future projections point at a significant increase in incidence, burden, and cost annually to >51,000 individuals and prevalence of >1.5 million cases by 2030 and an estimated annual cost of $3.8 billion in Australia. Specific considerations have to be given to priority and vulnerable populations, including elderly and Indigenous populations. These groups suffer disproportionately from disease burden, hospitalisations, non-traditional risk factors, and delays in uptake of Guideline-Based Medical Therapies (GBMTs).

## 3. Barriers in Interpreting/Accessing Clinical Evidence

Clinical trials represent the most rigorous method for assessing the effectiveness of novel treatments relative to established interventions with regard to specific outcomes. As such, well-conducted clinical trials possess the potential to exert a notable impact on patient care, and their design and execution should aim toward achieving this objective. However, the utility of clinical trials in knowledge translation applications is often undermined by the relatively few studies that render a meaningful contribution to patient care [3]. Such deficiencies often arise from limitations related to the selection, collection, and reporting of trial outcomes. Indeed, many trials report outcomes that inadequately reflect real-world settings and concerns, thereby rendering evidence generation difficult or unfeasible and undermining research translation efforts. These issues comprise a series of complexities, including the use of surrogate, composite, and subjective endpoints, limitations in accounting for patient perspectives, publication and reporting biases, such as inadequate assessment of adverse events, and the use of relative measures instead of more informative absolute outcomes, misleading reporting, multiplicity of outcomes, and insufficient core outcome sets [3,5].

Therefore, the strong internal validity (rigid controls) of clinical trials is at the expense of reduced external validity or generalisability when results are administered in global populations, with characteristics outside the trial criteria [6].

## 4. Challenges to Data Collection and Processing

Without a doubt, there have been issues with applying clinical trial evidence to heart failure practice. We discuss these challenges in more detail.

### 4.1. The Limitations of Translating Clinical Trials into HF Practice

Translational methodology must account for all health system and patient factors that are real and important contributors to the (cost-)effectiveness of treatments or interventions. Within the existing rigour of clinical trials and treatment discoveries, it will probably be a long while before the right balance for greater generalisability can be adopted in trial design; secondly, changes can more easily be documented as usable data if we are able to standardise existing health system service factors through disease management taxonomies [7] and landmark post-trial processes of care in translational studies [3,8]. Both cited papers show that organised care is vital for improving outcomes of CHF and other diseases. Thirdly, in terms of patient factors, there are numerous publications on CHF that point to variations in results from trials and in the population, which are discussed below.

### 4.2. Incidental Post-Trial Population-Level Patient Factors in Congestive Heart Failure

Clinical trials tend to over-enrol white, male patients, with consistent underrepresentation of women, the elderly, and, as demonstrated in US-based clinical trials, people of colour, especially African American and Hispanic patients. This skewed applicability of trial results has created several issues in heart failure treatment:

1. Initial studies on the use of vasodilators in heart failure provided evidence to support the prognostic benefit of nitrate in combination with hydralazine for heart failure with reduced ejection fraction in the 1980s. They are still the recommended therapy in some circumstances. Accumulating evidence from the V-HEFT and V-HEFT1 studies showed a greater benefit of this combination in the African American cohort. This was then confirmed in the A-HEFT study [9]. Additional studies with ethnographic differences were noted, including a blood pressure trial using diuretics and several renin aldosterone-modulating agents such as lisinopril and losartan [10];
2. Further epidemiological studies in African American cohorts showed other racial differences, particularly a higher incidence of and some traditional risks for hypertension and modifiable cardiometabolic risk factors [11,12];
3. Population-based genome-sequencing studies identified racial differences in allele frequency and incidence of HF [13,14]. Insofar as this extends to Black women, this population exhibits a higher incidence of peripartum cardiomyopathy, which is also less responsive to angiotensin-converting enzyme inhibitors (ACEi) and β-blockers [15]. A relative deficiency of natriuretic factors, a higher prevalence of salt sensitivity and sodium retention, as well as low physiological renin and aldosterone levels have treatment implications [16,17,18];
4. Based on the evidence outlined above, a combination of modifiable and non-modifiable factors must consider acquired and modifiable socioeconomic barriers to health equity [18]. It is at this juncture that we can start to appreciate the complexity of CHF and achieving equitable outcomes at the population level.

Controlled and supporting trials either screen out (exclude) or buffer these differences. However, appreciating that the level of evidence needed to answer our questions is very high, the onus now is to prescribe widely, replicate guidelines, and create a mechanism to identify the outcomes. If we are to take the example of socioeconomic and demographic factors, these may not be specific for any ethnicity. There are multiple levels of influence of racial and socioeconomic disparities on CHF epidemiology and outcomes. Additionally, models of care that improve these disparities must target layers of sociological, physiological, and jurisdictional constraints. Taking into account these factors when analysing the subjective and objective data included in a single study is important in these situations.

### 4.3. Patient-Reported Outcomes in Heart Failure

Patient-Reported Outcome Measures (PROMs) are used to measure patient experiences with disease and treatment, allowing for a deeper understanding of treatment impact beyond clinical endpoints [5]. PROM methods, such as questionnaires, are used in clinical trials or other clinical settings to help better understand a treatment’s efficacy or effectiveness. The use of digitised PROMs, or electronic patient-reported outcomes (ePROMs), is on the rise in today’s health research setting [6]. 

PROMs, in addition to being used in clinical trials, have diverse applications in clinical practice, clinical research, quality improvement, and policy development. They offer an efficient and standardised way to gather information on complex outcomes, such as daily functioning, while contributing to person-centred care and enhancing clinician–patient communication [19].

In clinical consultations, PROMs serve several purposes:

(i) Assisting patients in discussing concerns with their clinicians, such as changes in mental health during the course of treatment;
(ii) Sensitising patients to health issues, including symptoms related to their underlying health conditions and treatments that they may not have initially considered discussing with their clinicians;
(iii) Aiding in the identification of health issues that may require further investigation and management;
(iv) Facilitating the tracking of health outcomes over time;
(v) Allowing for comparisons between an individual patient’s outcomes and those of other patients with similar health conditions;
(vi) Supporting shared decision-making by providing information on the effects of different treatments on patient-reported outcomes.

Overall, PROMs extend beyond their role in clinical trials and have meaningful applications in various healthcare settings, enabling better patient care, improving patient–clinician encounters, and enhancing perceived control over health and informed decision-making [20]. Their utility in CHF, especially in clinical translation, has had stumbles; thus, their bench-to-bedside translation in CHF requires well-credentialled, skilled mixed-methods research teams. 

In heart failure patients, a number of PROMs are being used. For example, the New York Heart Association (NYHA) Classification is a good clinical tool; however, it is not sensitive for longitudinal research purposes. More reliable tools include the Minnesota Living with Heart Failure (MLHF) questionnaire and the Kansas City Cardiomyopathy Questionnaire (KCCQ).

In a study of 1037 Spanish CHF patients, the KCCQ showed CHF patients have a worse quality of life than the general Spanish population and patients with other chronic diseases, the predictors being the female sex, older age, comorbidity, advanced symptoms, and recent hospitalisation [21]. In the same year, [22] reviewed 19 PRO tools. Moreover, PROMIS + HF are increasingly being used; a recent study by Ahmed et al., 2022, developed and performed an initial validation of the PROMIS + HF-27 and PROMIS + HF-10 profiles, which successfully produced increased overall health, physical health, mental health, and social health summary scores [23]. 

In light of the recent FDA qualification of the KCCQ as a clinical outcome assessment for HF, the KCCQ provides good content, construct validity, and sensitivity to clinical change [24]. However, according to Chew et al., 2022, in terms of the validation of PROMs in CVDs, only a small minority of patients have been reported in the validation of all FDA-recommended psychometric properties. Given the use of PROMs to guide FDA approvals of drugs and devices in CVDs, there is an essential need for better observance of quality standards in PROM validation [25]. 

### 4.4. Utilising Data

Keeping in mind the above-mentioned limitations, particular attention should be paid to digital technology privacy and health privacy protections, which are a part of healthcare policy and legislation. Thus, the following questions arise: What kind of data can be used and what can they be used for?

In response to the first question, usable data can be divided into performance measures [6], performance standards [26], process of care [27], and audits and evaluations [28]. Large public and private hospitals are the most likely to align with these standards, as they are both required and resourced to do so. These data are also the most accessible. Nonetheless, most of these data will usually inform retrospective studies and, at best, may be help generate hypotheses. There are a range of emerging data wearables, apps, IoT, AI, VR, and AR, that could transition from an ambulatory non-clinical role to a clinical one. There are many barriers to this, largely related to the rigidness of health policy and legality, beyond their actual benefits. However, they can always be integrated within an audited, research ethics oversight and quality assurance role. 

The response to the second question is novel and requires an exploration of the purpose and processes of research undertakings. For data to be utilised in real-time prospective studies, it will require the standardisation of the collected performance measures and quality assurance measures to ensure operator variability and reliability are factored in. Often, there may be duplication of tests if patients are treated clinically and then go on to be enrolled in a trial. Thus, there are several issues here: firstly, transferring (or calibrating) the standards of large hospitals to specialty community providers, and secondly, standardising routine data to a clinical and research standard. Not to be forgotten in these discussions are the *technical challenges* and standards from an informatics perspective of integrating data from multiple sources (home vs. clinics) and maintaining a high quality of care. 

## 5. Building Models of Care for Community Hubs with System-Wide Performance Measures

There are only a few successful models of community CHF hubs that provide ambulatory tertiary-level care, including absorbing some subacute care, comparable to tertiary care [29]. Doing so supports ambulatory outpatient care, early post-hospital discharge care, and triage capabilities, which in turn significantly reduces readmissions and cost and improves chronic disease outcomes [19]. This is thus a domain of innovation, where new data can be generated. Importantly, this system-wide approach acknowledges that the patient journey and health system continuum are individual building blocks which by themselves are not constitutive of a system of care. Access issues can be negated by health providers by addressing gaps in the security and privacy of patients in their care at the level of individual organisations; by doing so, resourcing for collaborative team care with care coordination and cost-effectiveness can become a viable and potentially answerable research question.

The impetus for a system-wide approach to standardise performance measures is highlighted in the chronic care model (CCM) [5,30,31]. It has been almost three decades since the CCM highlighted that chronic patient care requires a multidisciplinary care team to achieve the greatest improvement in health outcomes. This model requires a complex process to coordinate and maintain health service expertise and skill, patient support and education, team-based care planning and delivery, and enhanced health information systems and registries, at the highest clinical levels. Adding in how data are stored and ensuring their quality and use thus rightfully remains an enormous challenge.

It is also important to note that while pharmaceutical and device treatments have achieved the highest levels of evidence in CHF guidelines, chronic disease self-management (CDSM) has been demoted due to a lack of evidence [32]. It is no surprise that in well-resourced health jurisdictions, multidisciplinary tertiary institution-based acute care model works: it generates large quantities of useful data for research, which contributes to the traditional model of CHF care. Much of health service research has been conducted under this framework and exploring established major adverse cardiovascular event (MACE) outcomes as endpoints. Generating broader data requires mixed methods, including a qualitative aspect and more heterogenous populations, which so far have been excluded from clinical trial enrolment criteria. When looking at broadening the pool of data, we note that doing so may benefit health service research. This benefit may have greater value for ancillary treatments for CHF like CDSM, and they can be studied from the perspective of their impact on MACEs and their contribution to cost-effectiveness. Unfortunately, the use of this type of non-traditional data in research is challenging; for example, how can legislative issues on privacy and sharing large amounts of data be meaningfully overcome? There remains a gap in the availability and quality of real-world community data that researchers can interrogate. 

## 6. Framework for Facing These Challenges

In this section, we outline several strategies to build on what has traditionally worked and to bridge current gaps in planning for the future. 

### 6.1. Adopting the Chronic Care Model (CCM)

**Figure 1:**
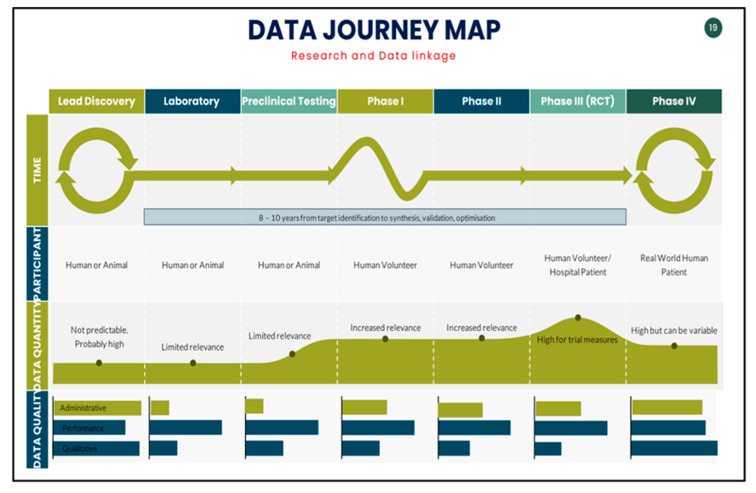
Data journey during research phase and post-trial phase.

Despite the challenges outlined above, we should at least focus on what works. Data collected from chronic care hubs at a research level can be used for further studies. The question here is how much can we bring this standard towards optimising routine care data and thus increasing the pool of usable research data? In Figure 1, we note that the largest component of health research data is from phase IV, which are essentially real-world data.

### 6.2. Improving Outcome Measures

Much progress has also been made in protecting patients’ data [33,34,35], while acknowledging more will come in the future [6]. Similarly, a lot is known on CHF pathophysiology [35]. However, there are gaps in knowledge on CHF and on improving this topic due to health data and privacy constraints. However, clear-cut performance areas can be identified, and to narrow down larger issues, high-value measures should be recorded at the highest standards. For example, it could help to address three important areas. The first is the epidemiology of heart failure with preserved ejection fraction (HFpEF). It represents 50% of new CHF cases, predominantly in older patients (>65 years), and faces multiple research gaps, as was already recognised in the Framingham trial many decades ago. The second is achieving a heterogeneity of MACE outcomes at the population level, and the third is reducing readmissions, as they are the most important contributor to CHF costs [36]. 

### 6.3. Funding Community-Based Health Hubs

All these topics can be researched by consistently investing in community chronic disease hubs, which will act as a sieve for these cases. Should data standards be high, this will mimic the ability to perform large population studies, such as the Framingham Heart Study, Olmsted County Study, Multi-Ethnic Study of Atherosclerosis (MESA), and others. Most vitally, it will give us access to non-traditional population data, which are deficient in systematic reviews that explore CHF epidemiology [8,36]. Thus, if we are to build on what works, we can accept two points: firstly, the acute care model has been successful, but it requires a concentration of resources; secondly, the chronic care model works best when the various arms of the health continuum are aligned for ambulatory care. Community-based health hubs, as satellites for clinical and research data, have issues as a concept, the most important of which is that in Australia, there are no funding models for this. The benefits include better patient access, experience, and outcomes; identification and management of CHF risk; a coordinated cardiac system of care; and effective and innovative cardiac services. It is hoped that, from a research data perspective, these hubs will add three dimensions:

(i) New data—generate usable data where previous data were of poor quality;
(ii) Scarce data—bring patient data together to create a larger volume of more sociodemographically diverse data with access to regional areas;
(iii) Traditional data—utilise existing data for retrospective, real-time, and prospective studies.

## 7. Conclusions

Health data are central to research in complex multidisciplinary chronic disease care. The example we have cited here is congestive heart failure (CHF). This area is topical as guideline-derived medical evidence is robust, while trial outcomes and selective community translational studies are well documented. The next steps in approaching CHF as a chronic disease are improving outcomes of broader groups of patients and reducing healthcare costs. What has become clear is that delivering guideline-derived care will improve outcomes. This gap in translational research requires access to a broad range of health data. In this review, we have discussed the challenges of this. We also raise observations on developments in health technology platforms that address technical challenges related to the usability and integration of data and collaborative links with the health service industry. This review aims to encourage discussion on this topic, break silos, and find an equilibrium between health and technology.

## Data Availability

Not applicable.
